# Recreating the Motion Trajectory of a System of Articulated Rigid Bodies on the Basis of Incomplete Measurement Information and Unsupervised Learning

**DOI:** 10.3390/s22062198

**Published:** 2022-03-11

**Authors:** Bartłomiej Nalepa, Magdalena Pawlyta, Mateusz Janiak, Agnieszka Szczęsna, Aleksander Gwiazda, Konrad Wojciechowski

**Affiliations:** 1 Department of Engineering Processes Automation and Integrated Manufacturing Systems, Faculty of Mechanical Engineering, Silesian University of Technology, Konarskiego 18A, 44-100 Gliwice, Poland; 2 Department of Computer Graphics, Vision and Digital Systems, Faculty of Automatic Control, Electronics and Computer Science, Silesian University of Technology, Akademicka 16, 44-100 Gliwice, Poland; magdalena.pawlyta@polsl.pl (M.P.); agnieszka.szczesna@polsl.pl (A.S.); konrad.wojciechowski@pjwstk.edu.pl (K.W.); 3 Polish-Japanese Academy of Information Technology, Koszykowa 86, 02-008 Warsaw, Poland; mateusz.janiak@pjwstk.edu.pl

**Keywords:** IMU sensors, industrial robots, Denavit-Hartenberg notation, ICA algorithm, parameter estimation

## Abstract

Re-creating the movement of an object consisting of articulated rigid bodies is an issue that concerns both mechanical and biomechanical systems. In the case of biomechanical systems, movement re-storation allows, among other things, introducing changes in training or rehabilitation exercises. Motion recording, both in the case of mechanical and biomechanical systems, can be carried out with the use of sensors recording motion parameters or vision systems and with hybrid solutions. This article presents a method of measuring motion parameters with IMU (Inertial Measurement Unit) sensors. The main assumption of the article is to present the method of data estimation from the IMU sensors for the given time moment on the basis of data from the previous time moment. The tested system was an industrial robot, because such a system allows identifying the measurement errors from IMU sensors and estimating errors basing on the reference measurements from encoders. The aim of the research is to be able to re-create the movement parameters of an object consisting of articulated rigid bodies on the basis of incomplete measurement information from sensors. The developed algorithms can be used in the diagnostics of mechanical systems as well as in sport or rehabilitation. Limiting sensors will allow, for example, athletes defining mistakes made during training only on the basis of measurements from one IMU sensor, e.g., installed in a smartphone. Both in the case of rehabilitation and sports, minimizing the number of sensors allows increasing the comfort of the person performing a given movement as part of the measurement.

## 1. Introduction

This article presents the problem of re-creating the motion of a system consisting of articulated rigid bodies. The motion was reproduced on the basis of data from IMU (Inertial Measurement Unit) sensors-angular velocity and linear acceleration. The mechanical parameters measured from the IMU sensors made it possible to calculate the torsion angles of individual members, i.e., the necessary parameter to re-create the movement of a given system. Measurement errors of IMU sensors were determined on the basis of calculating the difference between the values of parameters from IMU sensors and the measurement from the VICON marker vision system, which was taken as the reference measurement. 

The main goal of this article is to define the minimum number of IMU sensors that would allow re-creating the motion of a system consisting of articulated rigid bodies. The presented minimization algorithm is ultimately used in biomechanical systems, in particular in the reconstruction of human movement during sports activities and in the rehabilitation process. In this article, man has been replaced with an industrial robot. The purpose of changing the measuring system is to be able to use a different reference measurement, which in the case of an industrial robot are encoders. The encoders have a higher sampling frequency in relation to the marker vision system and lower susceptibility to external factors (e.g., the level of system illumination).

### State of Art

In the problem of data acquisition from systems consisting of articulated rigid bodies, the main sub-problems can be distinguished:

(a) the method of dividing the system into rigid bodies and the calculation method,
(b) selection of the measurement method,
(c) (optional) placement of the elements of the measurement system on the site and execution of the measurement,
(d) (purpose of this work) minimizing the elements of the measurement system on the site.

The method of dividing an object into rigid bodies depends mainly on the structure of the system under consideration, in which the connections between individual rigid bodies are also taken into account and certain limitations concerning the mobility of a given joint are defined. An example of dividing an object into rigid solids articulated are industrial robots [1,2,3,4,5] and humanoid robots [6]. Other examples are biomechanical systems, especially humans. In the case of humans, unlike robots, it is more often used to impose additional constraints (receiving degrees of freedom) that modify the original system, which can be observed in skiing, where the ankle joint is immobilized by a ski boot [7]. Therefore, human models are prepared depending on the movements performed in a given environment. The basic activities that can be distinguished are walking [8,9], swimming [10], skydiving [11]. 

The next stage, after dividing the system into rigid bodies and defining connections between solids and additional constraints, is to define kinematics or dynamics equations in the form of a simple or inverse task [1,2,3,4,5]. The result of the calculations is to obtain, among others, angles between individual members and the location of individual points in a given coordinate system. The description of the model can also be realized using the Denavit-Hartenberg notation together with the Newton-Euler equations [12,13,14,15,16,17,18,19,20] or the algebra of quaternions and dual quaternions [21,22,23,24,25].

There are two sub-problems in the problem of human movement acquisition: the first concerns the acquisition of human movement in a controlled laboratory environment using, for example, MoCap technology or pressure platforms [26,27,28,29,30,31,32,33], the second in uncontrolled conditions of everyday life, e.g., with the use of IMU sensors and shoes pressure inserts [25,34,35,36,37]. The selection of the test environment does not affect the defined model of the rigid body system, while the measurement technique and the time of the measurements are changed. The main goal is to determine the angles between individual members due to the importance of this parameter in diagnostics and rehabilitation. Techniques for human movement acquisition in a controlled laboratory environment are well developed, while in the everyday environment they are still under development and improvement. The main goal of work on measuring motion parameters in an uncontrolled environment is to minimize the number of sensors installed on a human body. A large number of sensors (often stuck to the body) may cause discomfort to the tested person, so the measurements may contain additional errors. The extreme case and, at the same time, the most comfortable for a human being is to perform the measurement with the use of only one IMU sensor, which would be in the smartphone of the person participating in the measurements.

The study attempts to analytically determine the relationship between the number of IMU sensors placed on individual rigid bodies and the possibility of determining the configuration of the kinematic chain on the basis of equations describing the relationships between individual members. So far, scientific works [38,39,40,41] have used the Kalman Filter or its extended form to filter measurement data on the basis of a known system model. The study [42] considered the case of minimizing the number of IMU sensors on the basis of known equations of the system model. The minimization of IMU sensors on the basis of the Kalman Filter was performed in [43]. The study investigated the pendulum model in which, on the basis of the Kalman Filter, the number of IMU sensors was minimized from three to one. However, in the work [43] each joint did not contain its own drive, and the input was applied to one of the components, therefore when considering a human system or a robot that has a drive (muscles or motor) in each joint, the algorithm presented in [43] will not be sufficient. In this study, the ICA (Independent Component Analysis) algorithm was used, assuming that one of the sensors from the IMU contains the resultant vectors (mixtures) of angular velocities and linear accelerations of all other IMU sensors.

## 2. Industrial Robot Model and Estimation

The FANUC ARC Mate 100iB industrial robot model was used in the conducted research. IMU sensors are mounted on the robot members as follows:

- IMU-1 → mounted on the axis of rotation of the robot base,
- IMU-2 → mounted halfway between the two joints of the other member,
- IMU-3 → mounted halfway between the two joints of the third segment.

**Figure 1:**
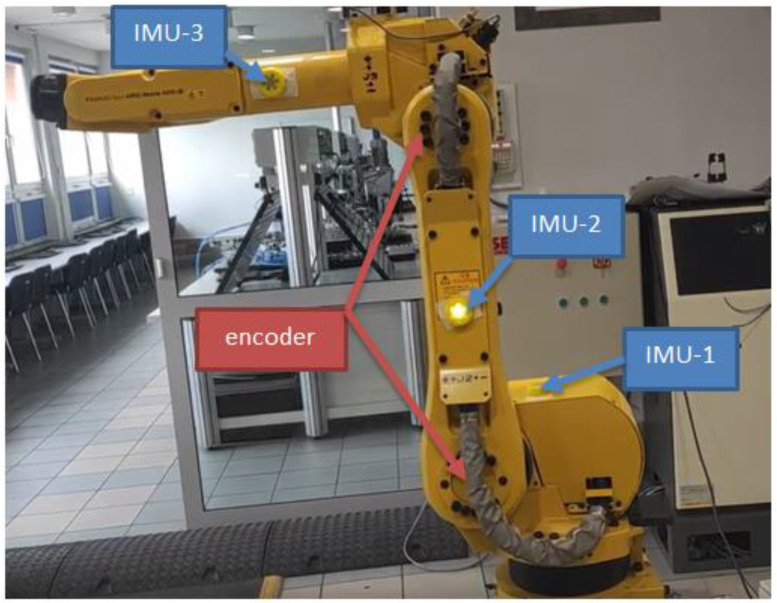
FANUC ARC Mate 100iB industrial robot with marked IMU sensors and encoders.

**Figure 2:**
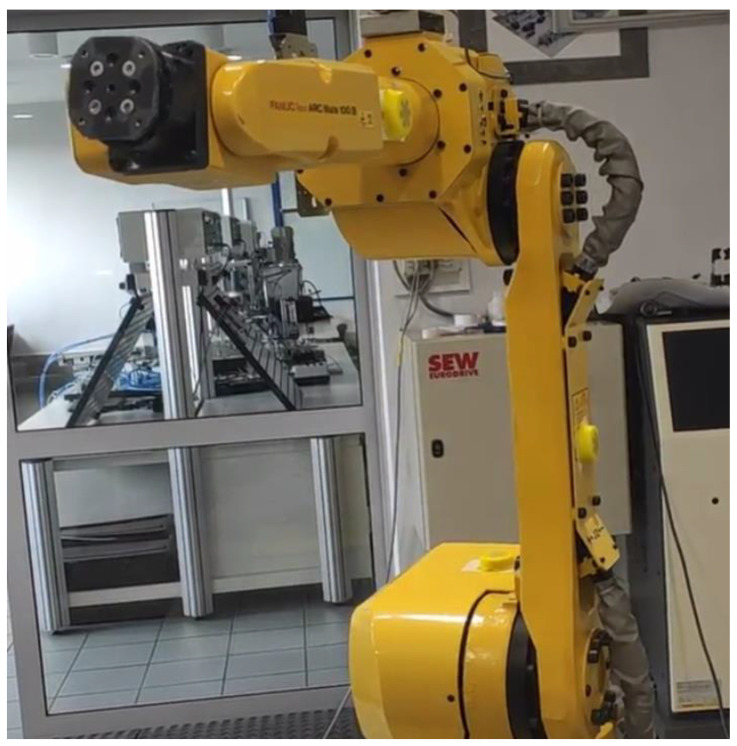
Placement of IMU sensors on the robot.

**Figure 3:**
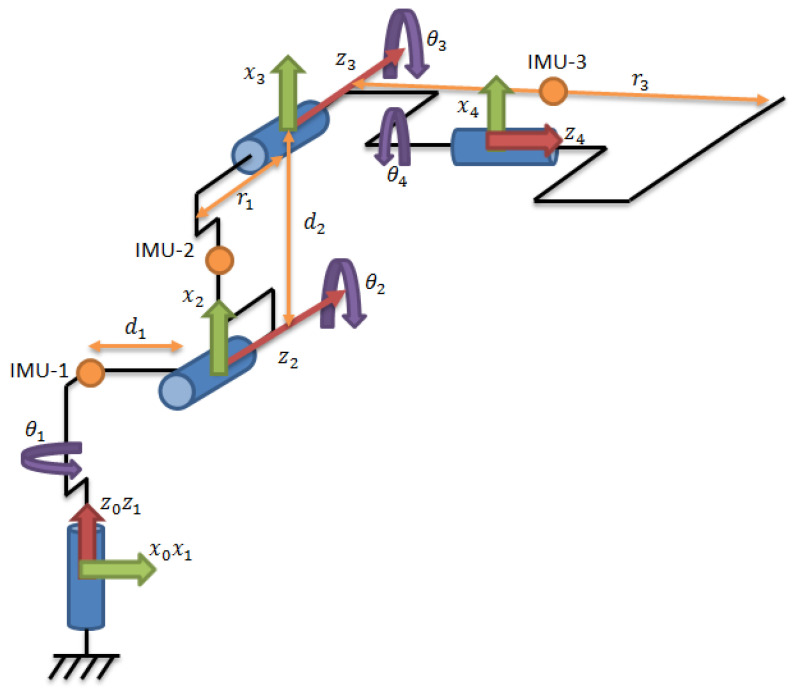
Partial kinematic diagram of the industrial robot (only 4axes for 3rd IMU-3 sensor) from Figure 1, containing a description of the coordinate systems only for the IMU-3 sensor.

Figure 1 and Figure 2 show the industrial robot with the placement of IMU sensors on individual members. Figure 3 shows a partial kinematic diagram of an industrial robot (description of the coordinate systems only for the IMU-3 sensor) containing the index numbers of the individual axes of the coordinate systems and the location of the IMU sensors.

### 2.1. Description of the Robot’s Kinematics

The robot kinematics equations can be written in several ways. This article will use the Denavit-Hartenberg (D-H) notation required to define the Newton-Euler equations. The first step is to define Table 1 of the D-H notation.

The following symbols can be distinguished in Table 1 [44]:

- $\theta_{i}$—the angle drawn around the Z axis,
- $r_{i}$—distance calculated between two coordinate systems, perpendicular to the X axis,
- $d_{i}$—distance calculated between two coordinate systems, perpendicular to the Z axis,
- $\alpha_{i}$—the angle drawn around the X axis.

Dependence on the general transformation matrix on the basis of which Table 1 was prepared [44]:

(1) Tii−1=Rx(αi−1)Dx(di−1)Rz(θi)Dz(ri)

Based on Table 1 containing the Denavit-Hartenberg notation, Newton-Euler equations can be written for angular velocities (measured by IMU sensors) [44]:

(2) ωi+1i+1=Rii+1ωii+θ˙i+1Z^i+1i+1

where:

- rotation matrix: Rii+1=[cosθisinθicosαisinθisinαi−sinθicosθicosαicosθisinαi0−sinαicosαi],
- angular velocity of the preceding term: ωii=[XiYiZi],
- angular speed of the axis drive (in the robot it is a servo motor): θ˙i+1Z^i+1i+1.

The speed value of the first robot segment is given by the relationship (the speed of the previous segment is equal to 0):

(3) ω11=θ˙1Z^11

The angular velocity of the second term is given by the relationship:

(4) ω22=[cosθ2sinθ20−sinθ2cosθ20001][W100]+[00W2]

where:

- $\left[\begin{array}{c} W_{1}\\ 0\\ 0 \end{array}\right]$—value of the angular velocity of the preceding term ω11 including the D-H notation (Table 1),
- $W_{1},W_{2}$—the assumed value of the own drive of the given axis (servo motor).

Equation for the third IMU sensor:

(5) ω33=[cosθ3sinθ30−sinθ3cosθ30001][−W1sinθ2−W1cosθ2W2]+[00W3]

Equation for the last IMU sensor:

(6) ω44=[cosθ40−sinθ4−sinθ40−cosθ4001][−W1sinθ2sinθ3+W1cosθ2cosθ3−W1sinθ2cosθ3−W1cosθ2cosθ3W2+W3]+[00W4]

where:

- $W_{1},W_{2},W_{3},W_{4}$—the assumed value of the own drive of a given axis (servo motor).

In the above equations, assume that:

(a) ωi+1i+1—it is the result of the measurement with the IMU sensor, i.e., the known value,
(b) ωii—speed of the term preceding the term in question,
(c) ${\dot{\theta }}_{i+1}$—own rotational speed of the considered element,
(d) $\theta_{i},\alpha_{i}$—torsion angles of individual members drawn around the Z axis and the X axis, (Table 1).

In the standard case, when IMU sensors are mounted on each part of the object, the values of ωi+1i+1 and ωii are known. Sensors are usually mounted at a characteristic point of each element (taking into account the geometrical characteristics of each term or at the centre of mass), therefore the value from the ${\dot{\theta }}_{i+1}$ is taken as a variable. Angle values from the $\theta_{i},\alpha_{i}$, are also unknown.

In order to achieve the goal of this article, i.e., to minimize the number of IMU sensors, it should be assumed that only the values from the variable ωi+1i+1 will be known. 

### 2.2. Modification of the ICA Algorithm and Experiment

By making the multiplication in relation (6) and extracting the equations for individual coordinates, it is possible to write an equation for the X coordinate:

(7) ω44x=−W1sinθ2sinθ3cosθ4+W1cosθ2cosθ3cosθ4−W2sinθ4−W3sinθ4

**Figure 4:**
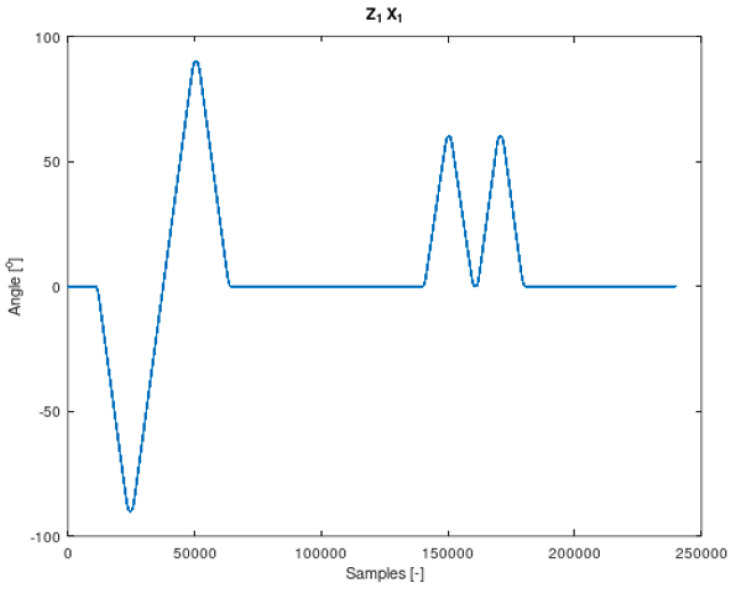
The angle set on the robot axis to which the coordinate system is assigned $z_{1}\;x_{1}$ in Figure 3. Data from FANUC robot encoders collected using the apparatus.

**Figure 5:**
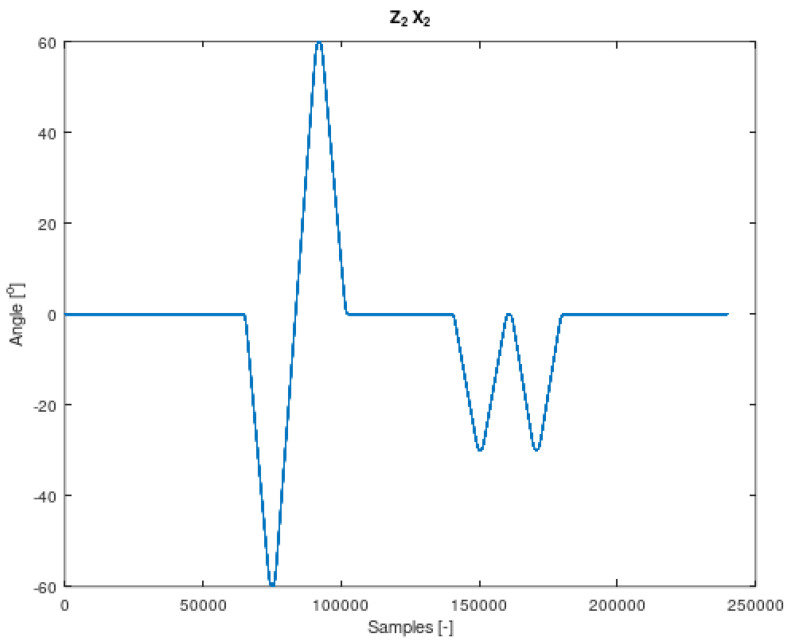
The angle set on the robot axis to which the coordinate system is assigned $z_{2}\;x_{2}$ in Figure 3. Data from FANUC robot encoders collected using the apparatus.

**Figure 6:**
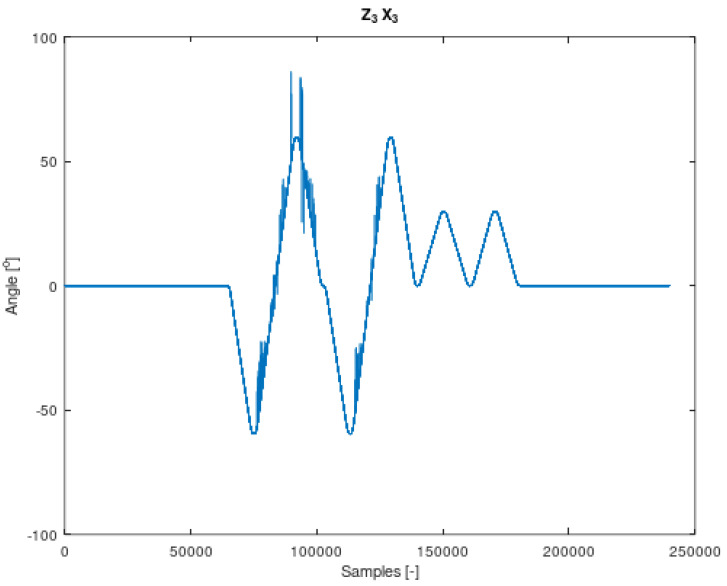
The angle set on the robot axis to which the coordinate system is assigned $z_{3}\;x_{3}$ in Figure 3. Data from FANUC robot encoders collected using the apparatus.

**Figure 7:**
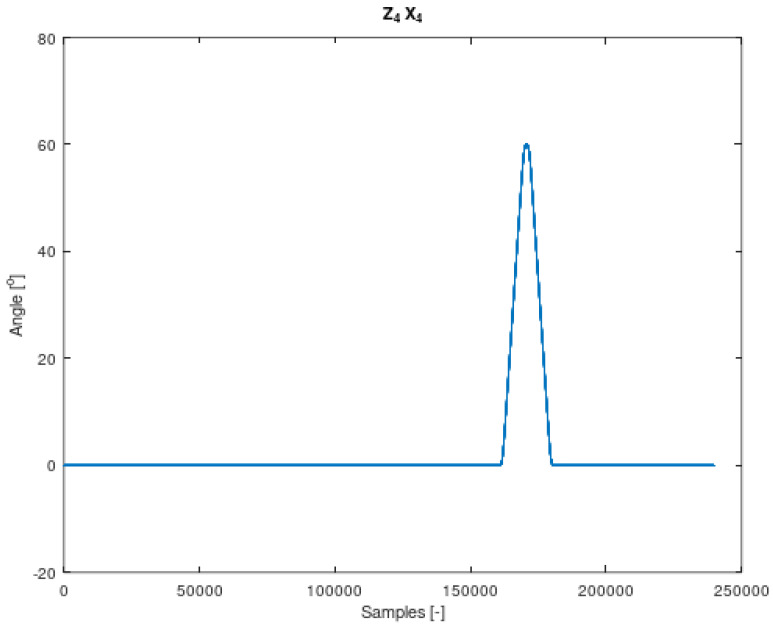
The angle set on the robot axis to which the coordinate system is assigned $z_{4}\;x_{4}$ in Figure 3. Data from FANUC robot encoders collected using the apparatus.

The components of Equation (7) are periodic functions. Equation (7) was extracted from Equation (6). It should also be noted that all the angles in Equations (6) and (7) are time-varying. The signal contained in Equation (7) is the resultant of signals on the remaining system members. It is therefore a mixture of signals from different IMU sensors. The analysed sensor, which contains the resultant values of angular velocities, is the IMU-3. Figure 4, Figure 5, Figure 6 and Figure 7 shows data diagrams from robot encoders showing the given angles on individual axes [45,46]. 

**Figure 8:**
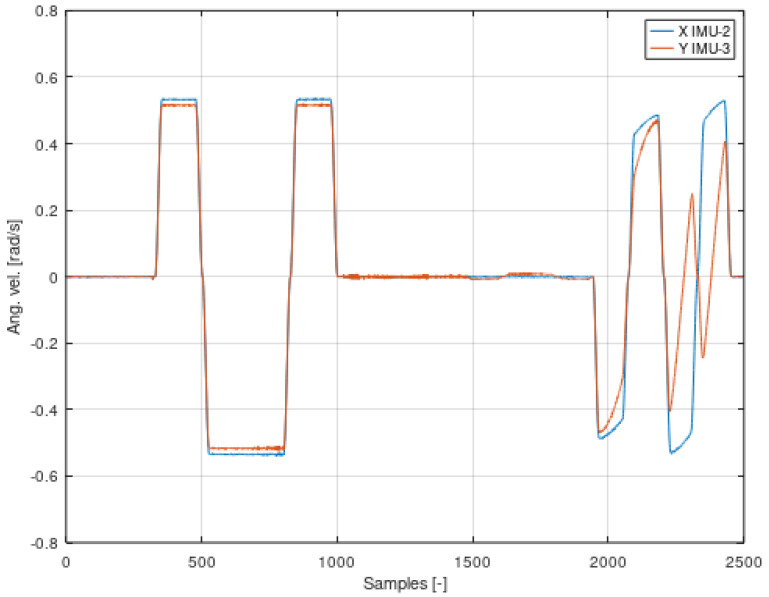
Summary of signals from the IMU-2 sensor of the X axis with the IMU-3 sensor of the Y axis.

An example of a mixture of signals is the diagram in Figure 8, which shows the summary of the signal for the X axis of the second IMU-2 sensor and the Y axis of the third IMU-3 sensor. The list of different axes (X and Y) results from the configuration of sensors mounted on the robot and shown in Figure 3.

The IMU-3 sensor on the Y axis saves data that was made and saved by the X axis of the IMU-2 sensor. The signal on the IMU-3 sensor is a composite of the signal generated by the unit on which the IMU-3 is attached and the signals recorded from the previous units. The *Y*-axis signal of the IMU-3 sensor is therefore a mixture of signals. The algorithm for solving problems related to mixing two signals and then their separation is the ICA (Independent Component Analysis) algorithm, given by the relationship [47]:

(8) x=AS

where:

- x—an input signal containing mixtures of signals,
- A—mixing matrix,
- S—source signals.

The ICA algorithm belongs to the set of unsupervised training algorithms. The algorithm includes computing the gradient of the equation containing the update of the matrix W (inverse of the matrix A). The update equation also includes the entropy function given as the activation function:

(9) U=tanh(x)

By multiplying the terms contained in Equation (7):

(10) ω33x=W1(−sinθ2sinθ3cosθ4+cosθ2cosθ3cosθ4)−W2sinθ4−W3sinθ4

The individual components of Equation (10) can be calculated on the basis of trigonometric identities and ultimately equation is equal to:

(11) $$\sin\theta_{2}\sin\theta_{3}\cos\theta_{4}=\frac{\cos\left(\theta_{2}-\theta_{3}-\theta_{4}\right)+\cos\left(\theta_{2}-\theta_{3}+\theta_{4}\right)-\cos\left(\theta_{2}+\theta_{3}-\theta_{4}\right)-\cos\left(\theta_{2}+\theta_{3}+\theta_{4}\right)}{4}$$

(12) $$\cos\theta_{2}\cos\theta_{3}\cos\theta_{4}=\frac{\cos\left(\theta_{2}-\theta_{3}-\theta_{4}\right)+\cos\left(\theta_{2}-\theta_{3}+\theta_{4}\right)+\cos\left(\theta_{2}+\theta_{3}-\theta_{4}\right)+\cos\left(\theta_{2}+\theta_{3}+\theta_{4}\right)}{4}$$

From Equations (11) and (12) it follows that each component of the angular velocity equation consists of the function sin() or cos(). When determining the entropy form for the ICA algorithm, it should be assumed that the trigonometric functions mentioned will be variable depending on one parameter x and compensated by appropriate constants in accordance with the dependencies:

(13) $$\begin{array}{l} \frac{\cos\left(\theta_{2}-\theta_{3}-\theta_{4}\right)+\cos\left(\theta_{2}-\theta_{3}+\theta_{4}\right)-\cos\left(\theta_{2}+\theta_{3}-\theta_{4}\right)-\cos\left(\theta_{2}+\theta_{3}+\theta_{4}\right)}{4}\\ \;=\frac{\cos\left(x\right)+a+\cos\left(x\right)+b-\cos\left(x\right)-c-\cos\left(x\right)-d}{4}=\frac{a+b-c-d}{4} \end{array}$$

(14) $$\begin{array}{l} \frac{\cos\left(\theta_{2}-\theta_{3}-\theta_{4}\right)+\cos\left(\theta_{2}-\theta_{3}+\theta_{4}\right)+\cos\left(\theta_{2}+\theta_{3}-\theta_{4}\right)+\cos\left(\theta_{2}+\theta_{3}+\theta_{4}\right)}{4}\\ \;=\frac{\cos\left(x\right)+e+\cos\left(x\right)+f+\cos\left(x\right)+g+\cos\left(x\right)+h}{4}\\ \;=\cos\left(x\right)+\frac{e+f+g+h}{4} \end{array}$$

(15) $$-W_{2}\sin\theta_{4}=-W_{2}\sin\left(x\right)+i\;\;\;\;\;\;\;\;\;-W_{3}\sin\theta_{4}=-W_{3}\sin\left(x\right)+j$$

where:

- a,b,c,d,e,f,g,h,i,j—compensation constants.

According to the ICA algorithm, the process of updating the inverse mixing matrix occurs through an entropy gradient. Therefore, the Equation (10) after the transformations contained in Equations (11)–(15) should be differentiated:

(16) ∂∂x(ω44x)=−W1sin(x)−(W2+W3)cos(x)

**Figure 9:**
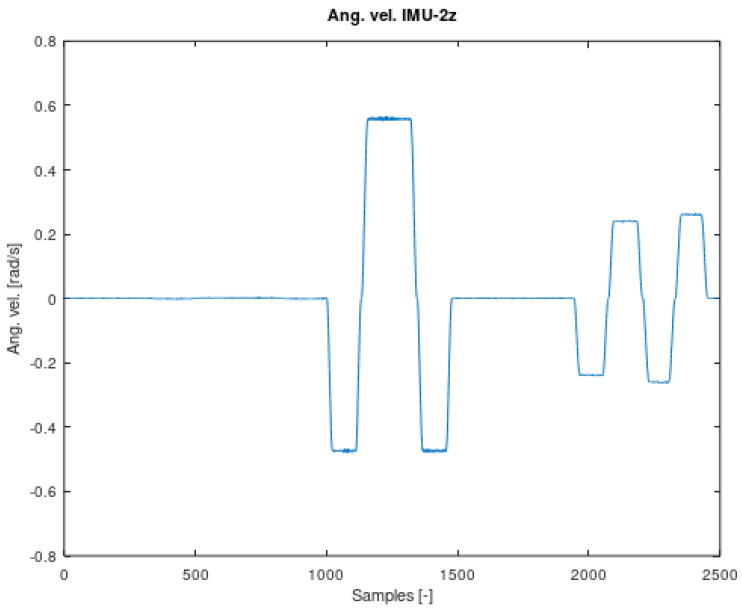
The angular velocity value for the axis Z IMU-2.

**Figure 10:**
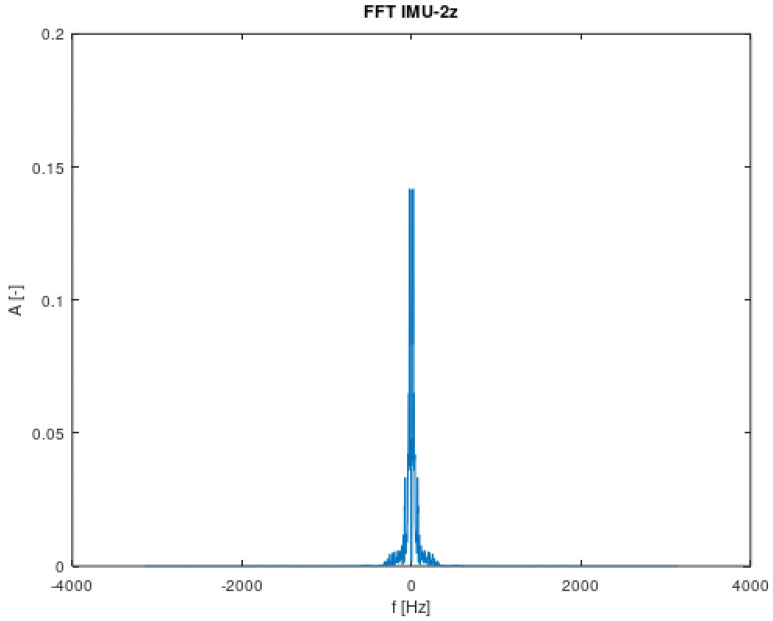
Fast Fourier Transform for IMU-2z.

As a result of the above considerations, Equation (15) will replace the standardly used tanh() function in the ICA algorithm. The proof of the thesis may be the juxtaposition presented in Figure 9 and Figure 10 comparing the angular velocity of the second IMU-2 sensor with its transform, and the results presented in the following figures.

Based on Figure 10, it can be concluded that the signal consists of many component signals. The amplitudes will be used for the analysis starting with the largest value and the number corresponding to the number of terms in the angular velocity equation. 

In the generalized case the Function (9) will be given by the relation:

(17) U=∂∂x(ωi+1i+1(x/y/z)(x))

An exemplary function element (containing a sin() or cos() function) is given by a relationship:

(18) $$A_{i}\cdot \frac{e^{ix}+e^{-ix}}{2}$$

where:

- $A_{i}$—the amplitude determined on the basis of the FFT.

**Figure 11:**
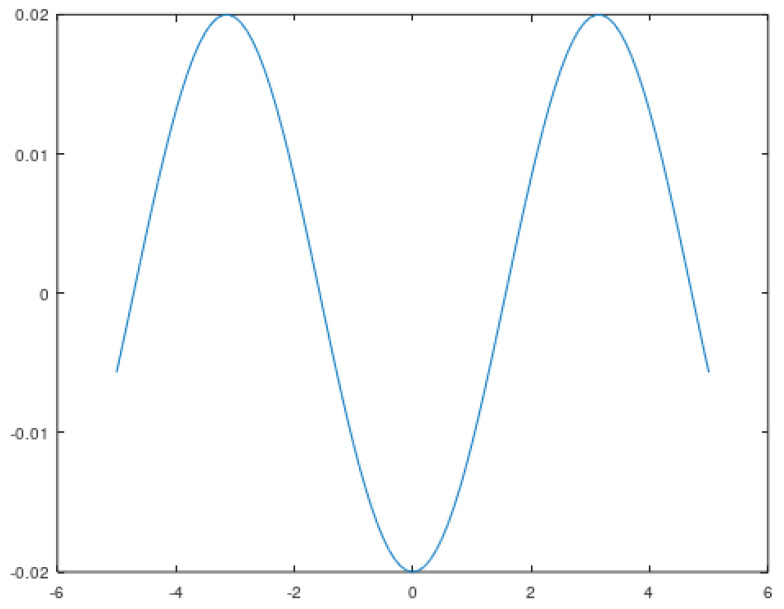
The ICA algorithm activation function determined on the basis of the dependence (9).

The form of the activation function is shown in Figure 11.

The set of signals shown in Figure 8 is a mixture of signals. The comparison, of these two signals, is resulted from the fact that the IMU sensors, from which the data are read, belong to other rigid bodies with separate coordinate systems. By analyzing Figure 8, it can be concluded that it is possible to distinguish two signals, i.e., a signal that was recorded on the second segment of the system and recorded on a sensor located on the third segment. One can also see a signal that comes only from the third term. The main task of this study is to select a sensor that contains the largest possible number of mixed data and to attempt identifying individual signals. 

**Figure 12:**
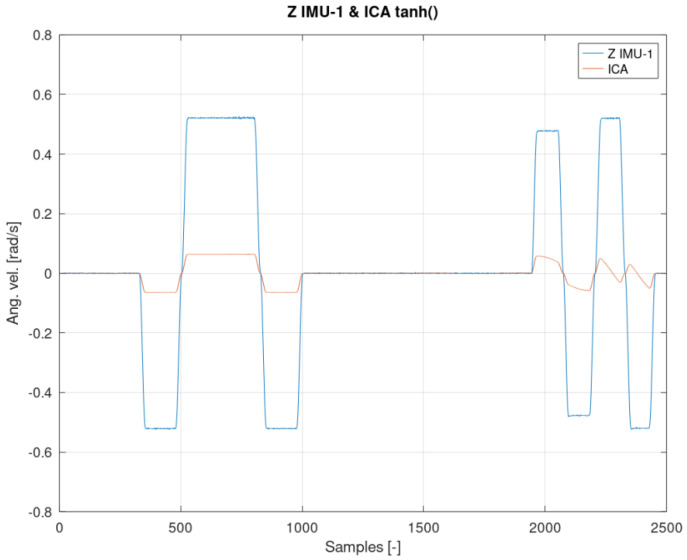
The result of the ICA algorithm on the basis of the activation Function (9).

**Figure 13:**
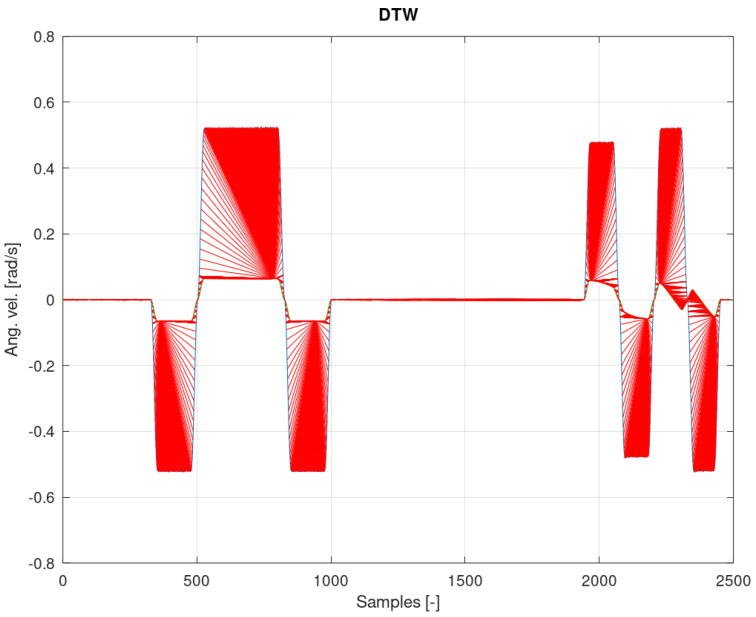
Analysis of the error from Figure 12 with the DTW algorithm.

**Figure 14:**
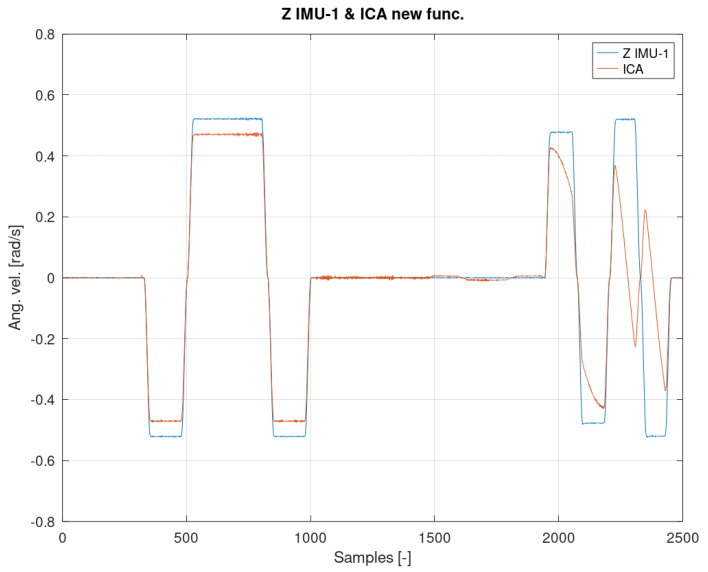
The result of the ICA algorithm on the basis of the activation Functions (17) and (18).

**Figure 15:**
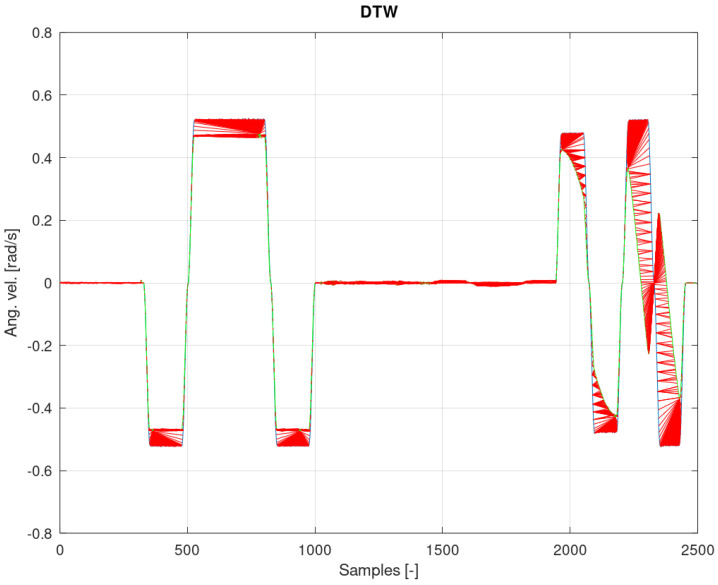
Analysis of the error from Figure 14 with the DTW algorithm.

**Figure 16:**
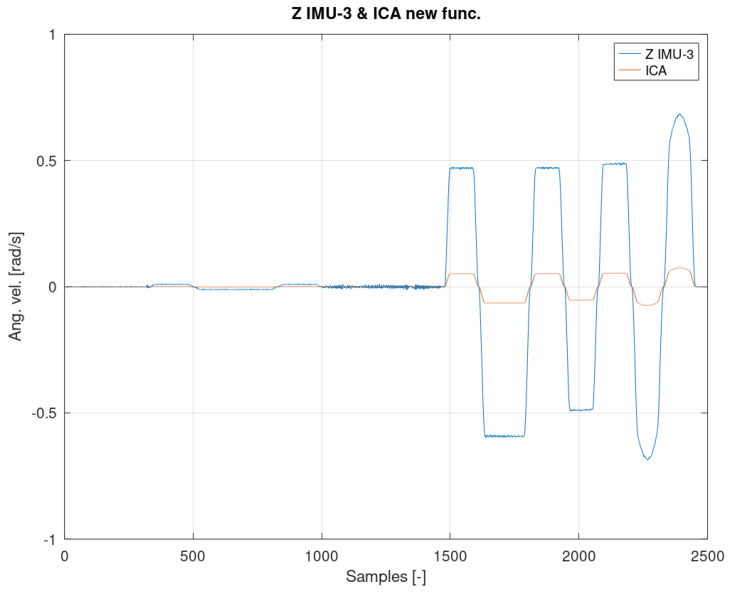
The result of the ICA algorithm on the basis of the activation Function (9).

**Figure 17:**
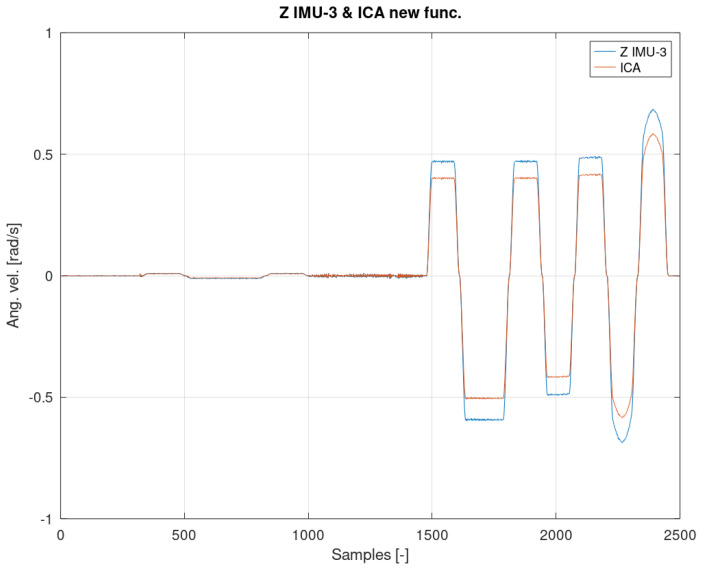
The result of the ICA algorithm on the basis of the activation Functions (17) and (18).

Figure 12, Figure 13 and Figure 14 show the measurement and estimation for the first IMU sensor, and Figure 15, Figure 16 and Figure 17 for the second IMU sensor. Figure 12 and Figure 16 show the signal estimation (Z axis IMU-1 and Z axis IMU-3, respectively) using the ICA algorithm along with the standard entropy gradient function with the given relationship (9). Figure 14 and Figure 17 show the application of the modified (Functions (17) and (18)) entropy gradient function. Figure 13 and Figure 15 present the analysis of the estimation error using the DTW (Dynamic Time Warping) method [48,49,50].

Based on the DTW algorithm (Figure 13 and Figure 15), the average measurement error was determined (tanh()//new function):

- axis Z IMU-1 → $0.046\frac{rad}{s}\;//\;0.0018\frac{rad}{s}$.

Based on the analysis of the above result, it can be concluded that the average error has been reduced by almost 96%.

## 3. Discussion

The article examines the possibility of re-creating the robot’s movement on the basis of incomplete measurement information. The aim of the research was to prepare an algorithm for estimating the parameters of sensors located on an object consisting of articulated rigid bodies in controlled laboratory conditions. The result of the research was an algorithm that could be used in uncontrolled working conditions.

The obtained test results were presented in relation to two entropy gradient functions. The use of the standard entropy gradient function (also called the activation function) is not efficient because this function is designed for the standard ICA algorithm and is based on changes in the probability function. In the case under consideration, the standard entropy gradient function was replaced with a derivative of the function describing the system model. The results presented in Figure 12, Figure 13, Figure 14, Figure 15, Figure 16 and Figure 17 confirm the correctness of the change in the entropy gradient function. The error in estimating the angle in the case of Figure 12, i.e., the system in which tanh () was used as a function of the entropy gradient, can be estimated, taking into account the maximum measurement error, at the level of about 20°. The use of the derivative of the function of the system model as a function of the entropy gradient resulted in the reduction of the measurement error to the level of about 6°. The reduction in error is significant, although it cannot be clearly defined as sufficient. The system is intended for sportsmen (both professionally and amateur) or people who are undergoing rehabilitation. The effectiveness of the method and the error rate must be assessed by specialists in the above-mentioned areas, i.e., trainers and doctors.

## 4. Conclusions

This article examines the possibility of reducing the number of measurement data, which would not result in a significant increase in the measurement error. The research was carried out on an industrial robot due to the possibility of using the reference measurement sources in the form of encoders. The tests were carried out with the use of IMU sensors mounted on the members of an industrial robot. The purpose of these tests was to verify the algorithm that could be applied to biomechanical systems. Verification of the athlete’s movement is important in terms of training, while in the case of rehabilitation it helps in the appropriate selection of exercises or loads.

The defined activation function allowed for a significant reduction of the estimation error, which allows for further work on the ICA algorithm along with the entropy gradient function, which can be used to estimate mechanical quantities. A significant reduction in error was obtained in the case of applying the entropy gradient function based on the object model than in the case of the standard entropy gradient function used in the classic form of the ICA algorithm, i.e., tanh().

In the next stages, an attempt should be made to use a neural network in the process of reducing the measurement error in the ICA algorithm.

## Figures and Tables

**Table 1:** Partial parameter table of D-H to IMU-3 notation.

No.	$\alpha_{i}$	$d_{i}$	$r_{i}$	$\theta_{i}$
1	0	0	0	$\theta_{1}$
2	$-\frac{\pi }{2}$	$d_{1}$	0	$-\frac{\pi }{2}$
3	0	0	0	$\theta_{2}$
4	0	$d_{2}$	$r_{1}$	$\frac{\pi }{2}$
5	0	0	0	$\theta_{3}$
6	0	0	0	$-\frac{\pi }{2}$
7	$-\frac{\pi }{2}$	0	$r_{3}$	$\theta_{4}$

## Data Availability

Measurement data from IMU sensors are available after contacting the correspondent author due to the need to report data sharing in the interdepartmental project.
